# Cell-type-specific role of P2Y2 receptor in HDM-driven model of allergic airway inflammation

**DOI:** 10.3389/fimmu.2023.1209097

**Published:** 2023-09-14

**Authors:** Dominik Schneble, Ahmed El-Gazzar, Zahra Kargarpour, Markus Kramer, Seda Metekol, Slagjana Stoshikj, Marco Idzko

**Affiliations:** ^1^ Department of Pneumology, Medical Center – University of Freiburg, Freiburg, Germany; ^2^ Department of Pulmonology, Medical University of Vienna, Vienna, Austria

**Keywords:** asthma, house dust mite, purinergic receptors, allergic airway inflammation, P2RY2

## Abstract

Allergic airway inflammation (AAI) is a chronic respiratory disease that is considered a severe restriction in daily life and is accompanied by a constant risk of acute aggravation. It is characterized by IgE-dependent activation of mast cells, infiltration of eosinophils, and activated T-helper cell type 2 (Th2) lymphocytes into airway mucosa. Purinergic receptor signaling is known to play a crucial role in inducing and maintaining allergic airway inflammation. Previous studies in an ovalbumin (OVA)–alum mouse model demonstrated a contribution of the P2Y2 purinergic receptor subtype (P2RY2) in allergic airway inflammation. However, conflicting data concerning the mechanism by which P2RY2 triggers AAI has been reported. Thus, we aimed at elucidating the cell-type-specific role of P2RY2 signaling in house dust mite (HDM)-driven model of allergic airway inflammation. Thereupon, HDM-driven AAI was induced in conditional knockout mice, deficient or intact for *P2ry2* in either alveolar epithelial cells, hematopoietic cells, myeloid cells, helper T cells, or dendritic cells. To analyze the functional role of P2RY2 in these mice models, flow cytometry of bronchoalveolar lavage fluid (BALF), cytokine measurement of BALF, invasive lung function measurement, HDM re-stimulation of mediastinal lymph node (MLN) cells, and lung histology were performed. Mice that were subjected to an HDM-based model of allergic airway inflammation resulted in reduced signs of acute airway inflammation including eosinophilia in BALF, peribronchial inflammation, Th2 cytokine production, and bronchial hyperresponsiveness in mice deficient for *P2ry2* in alveolar epithelial cells, hematopoietic cells, myeloid cells, or dendritic cells. Furthermore, the migration of bone-marrow-derived dendritic cells and bone-marrow-derived monocytes, both deficient in *P2ry2*, towards ATP was impaired. Additionally, we found reduced levels of MCP-1/CCL2 and IL-8 homologues in the BALF of mice deficient in *P2ry2* in myeloid cells and lower concentrations of IL-33 in the lung tissue of mice deficient in *P2ry2* in alveolar epithelial cells. In summary, our results show that P2RY2 contributes to HDM-induced airway inflammation by mediating proinflammatory cytokine production in airway epithelial cells, monocytes, and dendritic cells and drives the recruitment of lung dendritic cells and monocytes.

## 1 Introduction

Asthma is a chronic inflammatory disease (1) with an increasing prevalence affecting approximately 262 million in 2019, and it was responsible for 21.6 million disability-adjusted life years (DALYs) in the same year (2). Clinical symptoms are recurring breathlessness and wheezing accompanied by airway obstruction, bronchial hyperresponsiveness, and airway inflammation (3). Compelling evidence suggests asthma to be caused by a combination of genetic predispositions and exposure to environmental factors such as air pollution or allergens (4). The type 2 immune response causing an eosinophilic airway inflammation is initiated and maintained by airway epithelial cells along with dendritic cells (DCs) promoting innate lymphoid type 2 cell (ILC2) and Th2 cell activation (5). Activated ILC2s and Th2 cells secrete IL-4, IL-5, and IL-13, which mediates increased mucus secretion, airway smooth muscle contraction, allergen-specific IgE production, mast cell lung recruitment, and airway eosinophilia, all hallmarks of asthma pathogenesis (6).

Airway epithelial cells are the first point of contact of inhaled aeroallergens and secrete the DC maturation cytokines Thymic Stromal Lymphopoietin (TSLP) and Granulocyte-Macrophage Colony-Stimulating Factor (GM-CSF) (7). Pathogen-associated molecular pattern (PAMP) and damage-associated molecular patterns (DAMPs) bind their corresponding innate immune receptors driving allergic reaction by activating DCs (8, 9). In response to inflammatory, ischemic, and hypoxic conditions, nucleotides are released into the extracellular space. One of the earliest and most ubiquitous DAMPs is adenosine 5′-triphosphate (ATP), released at all inflammatory sites, where it activates nucleotide receptors that are called purinergic receptors (10).

Among the G-protein-coupled seven-transmembrane P2Y receptors is P2RY2, which has been implicated in the pathogenesis of various inflammatory disorders including allergic asthma by promoting inflammatory cell recruitment (11, 12). P2Y2 receptor is expressed in various epithelial cells (13–16), aortic smooth muscle (17, 18), and the brain (19, 20). Furthermore, P2Y2 receptor (P2RY2) expression has been shown in dendritic cells, eosinophils, B and T lymphocytes, macrophages, mast cells, monocytes, neutrophils, and NK cells (11, 21–27). P2RY2 is a well-known regulator of immune response and cell repair mechanisms (11, 28–31) that is activated by adenine and uracil nucleotides and triphosphates but not diphosphates (20, 32). In bronchial and intestinal epithelial, activation of P2Y2 receptor results in stimulation of chloride secretion and inhibition of NA^+^ transport (33–35). Hence, P2Y2 is a therapeutic target for cystic fibrosis (36). Moreover, it has been reported that P2Y2 receptor agonists enhance ciliary beat frequency and modulate mucin release in animals (33, 37, 38). Furthermore, the P2Y2-receptor-deficient mouse shows salt-resistant arterial hypertension (39). P2RY2 activation has been reported to be an initiator of inflammasome activation (40). We have previously shown that P2Y2 contributes to allergic lung inflammation by promoting migration of DCs and eosinophils (25). It has also been reported that activation of P2RY2 by eATP induces vascular inflammation and atherosclerosis (41).

As mentioned above, P2RY2 is predominantly expressed in immune cells (42), but its role in allergen-induced airway inflammation in a cell-type-specific manner has not yet been completely elucidated. In the current study, we aimed to identify the physiological and/or pathophysiological cell-type-specific role of P2RY2 in allergic airway inflammation. Here, we used conditional *P2ry2* knockout expressing cre-recombinase in different cell types in an acute HDM-induced model of airway inflammation. As a result, the ablation of *P2ry2* in epithelial cells, DCs, and myeloid cells alleviates AAI, whereas *P2ry2* deficiency in CD4-positive T cells did not exert an attenuating effect on allergic lung inflammation.

## 2 Materials and methods

### 2.1 Mice

Conditional *P2ry2*-floxed (*P2ry2^fl/fl^*) mice on a C57BL/6 background were generated by TaconicArtemis GmbH (Cologne, Germany). Two Cre recognition sequences (loxP sites) were inserted into intron 2 of the *P2ry2* genomic sequence to flank exon 3. Exon 3 includes the entire coding region of P2RY2 gene. The insertion of the LoxP site into the *P2ry2* gene facilitated Cre-mediated deletion of the entire *P2ry2* coding region.

To generate conditional knockout mice, *P2ry2^fl/fl^* mice were mated with Cre^+^ mice of the lines C57BL/6-Tg(Scgb1a1-cre)1Tauc (*Cct*-cre) MGI:3610310 (43), C57BL/6-Tg(Vav1-icre)A2Kio/J (*Vav*-cre) MGI:2449949 (44), C57BL/6-Lyz2tm1(cre)Ifo (*LysM*-cre) MGI:1934631 (45), C57BL/6-Tg(*Cd4*-cre)1Cwi/BfluJ (*Cd4*-cre) MGI:5317293 (46), or C57BL/6-Tg(Itgax-cre)1-1Reiz/J (*Cd11c*-cre) MGI:3763248 (47) mice in the animal facilities of Freiburg University in order to receive cell-type-specific conditional knockout mice. All of these mice are on a C57BL/6 background and were maintained on this background.

In *Cct*-cre-positive mice, cre-recombinase is mainly expressed in the epithelial cells of the proximal conducting airways due to the transcriptional control by the major Clara cell secretory protein (CCSP) promoter (48). *Vav*-cre-positive mice show cre-recombinase activity in cells of the hematopoietic compartment and endothelial cells (49). In *LysM*-cre positive mice, cre-recombinase is mainly expressed in cells of the myeloid lineage (45) but also occurs in epithelial cells (50). Cd11c-cre^+^-positive mice exhibit cre-recombinase activity mainly in conventional dendritic cells (47), whereas *Cd4*-cre-positive mice show cre-recombinase activity exclusively in CD4^+^ cells (51).

Four to six female mice, 6–9 weeks, were considered in each group. All strains were bred and housed under specific pathogen-free (SPF) conditions. Mice experiments were performed in accordance with the local ethic committee (G12-096).

### 2.2 Verification of cre-lox models

Epithelial cells, peripheral blood mononuclear cells (PBMCs), granulocytes, CD11c^+^ cells, CD4^+^ cells, bone marrow monocytes, and bone-marrow-derived macrophages from concerned strains were isolated, and P2Y2 receptor mRNA levels were measured by quantitative polymerase chain reaction (qPCR). Results were compared to cre-negative littermates (**Supplementary Figure S1**).

### 2.3 House dust mite extract induced airway inflammation

Anesthetized mice received three consecutive intratracheal (i.t.) administrations of 80 µL of Phosphate Buffered Saline (PBS) (Gibco, NY, USA) along with or without 100 µg house dust mite extract (HDM, Greer Laboratories, Lenoir, USA) at days 0, 7, and 14. Notably, the level of the endotoxin LPS in the commercial extract is 55,750 EU/vial (vial = 2.5 mL). The control group received PBS instead of HDM at days 0 and 7. All groups received HDM at day 14. After 72 h from the last HDM application, mice were either subjected to lung function measurement or sacrificed for bronchoalveolar lavage fluid (BALF) and mediastinal lymph node (MLN) isolation (25, 52–54). Subsequently, lungs were resected in Tissue-Tek optimal cutting temperature (OCT) compound (Sakura Finetek, CA, USA) for histological analysis. For BALF, the lungs were flushed three times with 1 mL of bronchoalveolar lavage buffer (PBS, 0.5 mM EDTA) each time. Usually, approximately 2 mL could be recovered. The three washes were pooled and then used for the flow cytometry and cytokine analysis. The bronchoalveolar lavage fluid obtained is centrifuged for 7 min at 8°C at 400*g*. The supernatant is then collected for cytokine measurements. Cell pellets were used for flow cytometry analysis. Cytokine concentrations in supernatants of BALF and MLN cells re-stimulated with HDM extracts (30 µg/mL) for 5 days at 37°C with 5% CO_2_ were determined using ELISA DuoSets (R&D Systems, USA) (55).

### 2.4 Invasive lung function measurement

Anesthetized mice were intubated with an 18-gauge catheter, placed in a whole-body plethysmograph (EMMS, Bordon, UK) and mechanically ventilated (Minivent 485, Hugo-Sachs, March, Germany) at a rate of 200 strokes per minute with a volume of 200 µL. Airway resistance was recorded by eDacq software (EMMS, Bordon, UK) via a trans-pulmonary pressure transducer (TPP 200, EMMS, Bordon, UK) connected to an amplifier interface unit (AAC 091, EMMS, Bordon, UK), while mice were exposed to increasing doses (0 mg/mL, 3 mg/mL, 10 mg/mL, 30 mg/mL, and 100 mg/mL) of nebulized methacholine (Aristo Pharma GmbH, Germany).

### 2.5 Generation of bone-marrow-derived monocytes and macrophage cells

Bone marrow (BM) were isolated from the tibia and femur of cre-positive and cre-negative mice by flushing the medullary cavity. After that, the cells were centrifuged and then resuspended in a red blood cell lysis buffer [ammonium chloride lysis buffer (150 mM NH_4_Cl, 10 mM KHCO_3_, 100 nM EDTA, dH_2_O, pH=7.4)] for 5 min on ice. Subsequently, 2×10^6^ bone marrow cells were cultured in 2 mL Roswell Park Memorial Institute (RPMI) 1640 medium (Gibco, USA) containing 10% fetal calf serum (FCS, Sigma-Aldrich, Germany), 1% penicillin/streptomycin (Gibco, USA), and in 20 ng/mL of murine macrophage colony-stimulating factor (m-CSF, Miltenyi Biotec GmbH, Germany) in an Ultra-Low Attachment six-well plate (Corning Incorporated, USA) at 37°C with 5% CO_2_. On day 5, the non-adherent cells were collected and centrifuged at 400*g* at 4°C for 10 min to generate bone-marrow-derived monocytes (56, 57). The adherent cells were plated in complete medium to generate bone-marrow-derived macrophage.

### 2.6 Generation of bone-marrow-derived dendritic cells

Bone-marrow-derived dendritic cells (BMDCs) were generated with recombinant human Flt3 ligand (rh FLt3L/CD135) (ImmunoTools, Germany). In brief, bone marrow cells were isolated from the indicated experimental animals as described above and were grown at a concentration of 1×10^6^ cells per well onto a 24-well plate in RPMI 1640 medium supplemented with 10% FCS, 1% penicillin/streptomycin, 50 μM 2-mercaptoethanol (Bio-Rad Laboratories, Germany), and 200 ng/mL rh FLt3 ligand. On day 6, the medium including 200 ng/mL rh FLt3 ligand was renewed. On day 9, the purity of bone-marrow-derived dendritic cells was >90% based on the proportion of the population expressing CD11c as determined by flow cytometry.

### 2.7 *In vitro* migration experiments with DCs and monocytes

Migration assay was performed as described before (25, 58). Briefly, 1×10^5^ cells/well in 100 µL medium was added to the upper compartment to each insert of a 24-well Transwell system with a pore size of 5 µm (Corning Incorporated, USA). To the lower compartment wells, ATP (Sigma-Aldrich, Germany) in different concentrations was added. Following 90 min of incubation at 37°C in a humidified atmosphere, cells from the upper compartment were removed, and migrated cells in the lower compartment were harvested and quantified by flow cytometry to determine the number of migrated cells. Only living F4/80^−^ cells for monocytes and CD11c^+^ cells for DCs were considered for calculation of migratory index. Results are shown as chemotactic index defined as the number of F4/80^−^ or CD11c^+^ cells in the lower compartments containing ATP divided by the number of F4/80^−^ or CD11c^+^ cells in the chamber containing medium only. Experiments were performed in duplicate.

### 2.8 Lung digestion

Resected lungs from *P2ry2^fl/fl^ Cct*-cre^+^ mice and cre-negative littermates were filled up with 1 mL dispase (Corning Incorporated, USA) and digested in Dulbecco’s Modified Eagle Medium (DMEM) containing 2 µg/mL collagenase (Sigma-Aldrich, Germany) and 0.001% DNAse (Serva, Germany). Anti-mouse CD31^−^ (Biolegend, CA, USA), anti-mouse CD45^−^ (Biolegend, CA, USA), and anti-mouse EpCAM^+^ (Thermo Scientific, Germany) cells were sorted with FACS Aria III (BD Biosciences, Germany). Subsequent qPCR was performed to evaluate P2ry2 expression.

### 2.9 Magnetic isolation of CD11c^+^ cells from lung digest

CD11c^+^ cells were isolated from the lung cell suspension with CD11c^+^ microbeads (Miltenyi Biotec, Germany), and subsequently, qPCR was performed.

### 2.10 Magnetic isolation of CD4^+^ cells from spleen

Spleens from *P2ry2^fl/fl^ Cd4*-cre^+^ and WT mice were excised and smashed through a 100-µm cell strainer (Corning Incorporated, USA). Cells were let rest for 10 min, and supernatants were collected. Subsequently, CD4^+^ cells were isolated from the cell suspension with CD4^+^ microbeads (Miltenyi Biotec, Germany).

### 2.11 Cell separation with polysaccharide separating solutions

A total of 500 µL of peripheral blood was taken from Vena cava of *P2ry2^fl/fl^ VaV*-cre^+^ mice, *P2ry2^fl/fl^ LysM*-cre^+^ mice, and cre negative littermates, supplemented with 50 µL 0.5M EDTA (Serva, Germany) and diluted with 500 µL of PBS. Cell separation was performed with polysaccharide separating solutions Pancoll (PAN-Biotech, Germany). After isolation, PBMCs and granulocytes were stimulated with LPS (300 ng/mL), and qPCR was performed to detect *P2ry2* expression.

### 2.12 Flow cytometry

Flow cytometry analysis of the BALF was performed as described previously in (59, 60). Briefly, BALF was performed with 3 × 1 mL of Ca^2+^- and Mg^2+^-free PBS supplemented with 0.1 mM EDTA. Red blood cells were lysed using ammonium chloride lysis buffer (150 mM NH_4_Cl, 10 mM KHCO_3_, 100 nM EDTA, dH_2_O, pH=7.4). BALF cells were then counted under a microscope and washed. 0.25×10^6^ to 1×10^6^ cells/mL was used in each sample. First, BALF cells were incubated for 10 min with an unlabeled rat anti-mouse CD16/CD32 antibody (Mouse BD Fc Block, BD Biosciences, Germany) to avoid unspecific FcγRIII and FcγRII binding. Subsequently, cells were stained with FITC conjugated rat monoclonal anti-mouse Ly-6G (Gr-1) (clone 1A8-Ly6g, dilution 1:50, Thermo Scientific, Germany), PE-conjugated anti-mouse CD193 (CCR3) (dilution 1:50, eBioscience, USA), APC-conjugated Armenian hamster monoclonal anti-mouse CD11c (clone N418, dilution 1:400, Thermo Scientific, Germany), and PE-Cy7-conjugated rat monoclonal anti-mouse CD3− (clone 17A2, dilution 1:50, Thermo Scientific, Germany) and with PE-Cy7-conjugated rat monoclonal anti- B220 (clone RA3-6B2, dilution 1:100, Thermo Scientific, Germany) antibodies in FACS buffer (PBS containing 2.5% FCS, 5 mM EDTA and 0.01% sodium azide). Cells were incubated for 30 min at 4°C in the dark, washed twice with FACS buffer, and then resuspended in FACS buffer and analyzed by FACS. Flow cytometry was done immediately after the completion of the staining protocol. To establish proper gating strategy, isotype Igs were used as controls. A flow cytometer with appropriate lasers and filters for signal detection was used. It was performed using a FacsCalibur flow cytometer (BD Biosciences, USA) using Cellquest version 3.3 (BD Biosciences, USA). Data analysis was done using FlowJo software (TreeStar Inc., USA). For gating strategy, please see **Supplementary Material and Methods.** Approximately 1×10^5^ was analyzed per sample. Murine cell frequencies are indicated in the plots in **Supplementary Material and Methods.**

### 2.13 Quantitative PCR

For quantitative PCR, Takyon Mastermix (Eurogentec, Germany) was used. All measurements were executed with LightCyler 480 (Roche, Germany). An annealing temperature of 59°C has been chosen for all reactions. All primers and dual-labeled probes were designed as described before (61). All sequences are available upon request. The reference gene (RG) utilized was ß2m. To calculate the percent RG values, the following formula was used: %RG = 100 × 2^(−Ct)^. The following formula was used for the calculation of the combined standard deviations of reference gene and gene of interest (GOI): SD = 100 × 2^(−DCt)^ × ((ln2 × SD_RG_)^2^ + (ln2 × SD_GOI_)^2^)^1/2^ (62).

### 2.14 Statistical analysis

For the calculation of the statistical significance of differences between study groups and controls, one-way ANOVA was applied, followed by Bonferroni comparison test. Two-sided Student’s *t*-tests were performed when indicated. Analyses were performed using GraphPad Prism v8 (GraphPad Software, USA). Differences were considered significant at *p* < 0.05 and highly significant at *p* < 0.005.

## 3 Results

### 3.1 *P2ry2* deficiency in alveolar epithelial cells mitigates HDM-induced airway inflammation

**Figure 1:**
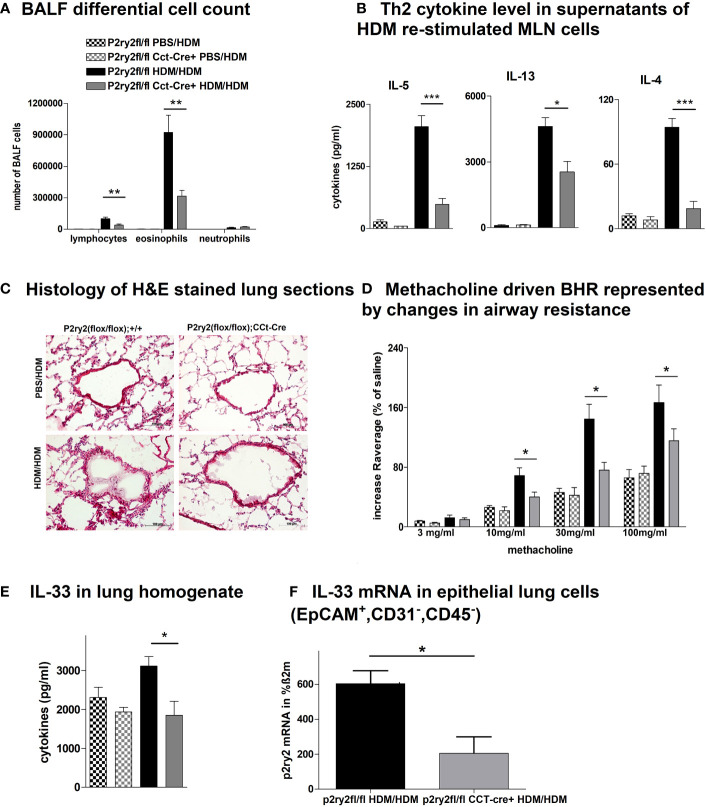
*P2ry2* deficiency in *P2ry2^fl/fl^ CCt*-cre^+^ mice decreases allergic airway inflammation. **(A)** BALF differential cell count. **(B)** Th2 cytokine concentrations in supernatants of HDM re-stimulated MLN cells. **(C)** Histology of H&E-stained lung sections obtained from *P2ry2^fl/fl^* CCt-cre^+^ mice and *P2ry2^fl/fl^* mice treated with HDM (magnification: 20× objective). **(D)** Methacholine-driven BHR represented by changes in airway resistance. **(E)** Level of IL-33 in the lung homogenate. **(F)** Relative IL-33 mRNA level in EpCAM^+^, CD31^−^, and CD45^−^ lung cells. Graphs show mean ± SEM (n = 4–6). *p < 0.05, **p < 0.01, ***p < 0.001, *P2ry2^fl/fl^ CCt-cre^+^* HDM/HDM vs. *P2ry2^fl/fl^* HDM/HDM.

To determine the protective role of *P2ry2* in epithelial cells in allergic airway inflammation mouse model, we sensitized *P2ry2^fl/fl^ Cct*-cre^+^ mice deficient for *P2ry2* on airway epithelial cells and in control littermate. Subjecting *P2ry2^fl/fl^ Cct*-cre^+^ mice to the HDM-induced model of airway inflammation resulted in an alleviated BALF eosinophilia (**Figure 1A**) and type 2 cytokine production of HDM re-stimulated MLN cells (**Figure 1B**). Histological analysis of H&E-stained lung sections showed decreased peribronchial inflammation in *P2ry2^fl/fl^* CCt-cre^+^ mice compared to *P2ry2^fl/fl^* mice treated with HDM (**Figure 1C**). To evaluate invasive lung function, airway resistance was recorded, and the results revealed a decrease in bronchial hyperreactivity (BHR) in response to increasing doses of nebulized methacholine compared to cre-negative littermates (**Figure 1D**). Furthermore, we found lower concentrations of IL-33 in the lung tissue homogenate (**Figure 1E**) and EpCAM^+^, CD31^−^, and CD45^−^ lung cells at mRNA level (**Figure 1F**). Altogether, these results suggest that the epithelial cells deficient in *P2ry2* show reduced HDM-induced airway inflammation in mice.

### 3.2 *P2ry2*-deficient mice in hematopoietic cell lineage exhibit an attenuated AAI phenotype

**Figure 2:**
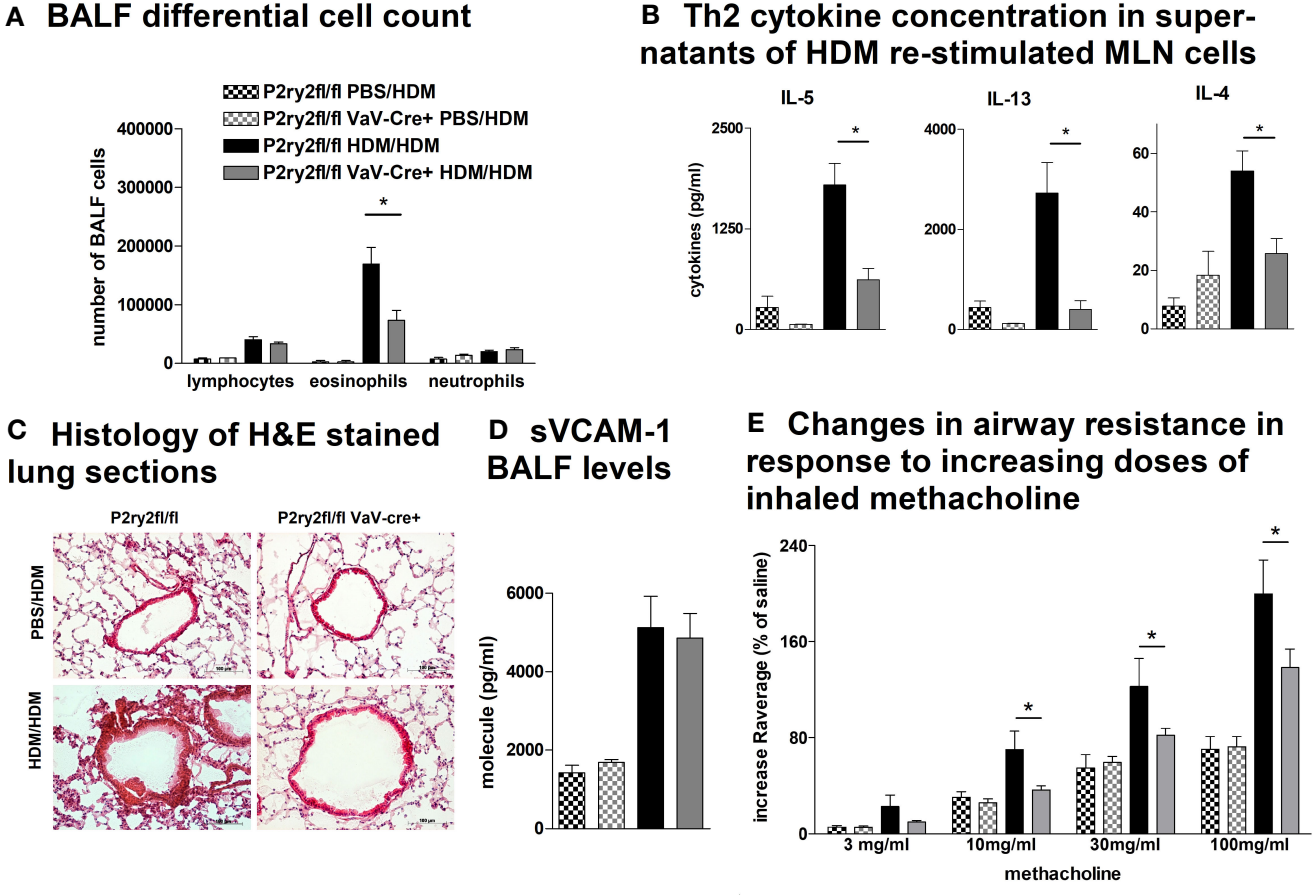
*P2ry2^fl/fl^ Vav*-cre^+^ mice deficient in *P2ry2* show less AAI than cre^−^ littermates. **(A)** BALF differential cell count. **(B)** Th2 cytokine concentrations in supernatants of HDM re-stimulated MLN cells. **(C)** Histology of H&E-stained lung sections (magnification: 20× objective) isolated from *P2ry2^fl/fl^ Vav*-cre^+^ and *P2ry2^fl/fl^* mice after HDM-challenge. **(D)** Changes in airway resistance in response to increasing doses of inhaled methacholine. **(E)** Concentration of soluble vascular cell adhesion molecule 1 (sVCAM-1) in BALF supernatant. Graphs show mean ± SEM (n = 4–6). *p < 0.05, *P2ry2^fl/fl^ Vav-cre^+^* HDM/HDM vs. *P2ry2^fl/fl^* HDM/HDM.

**Figure 3:**
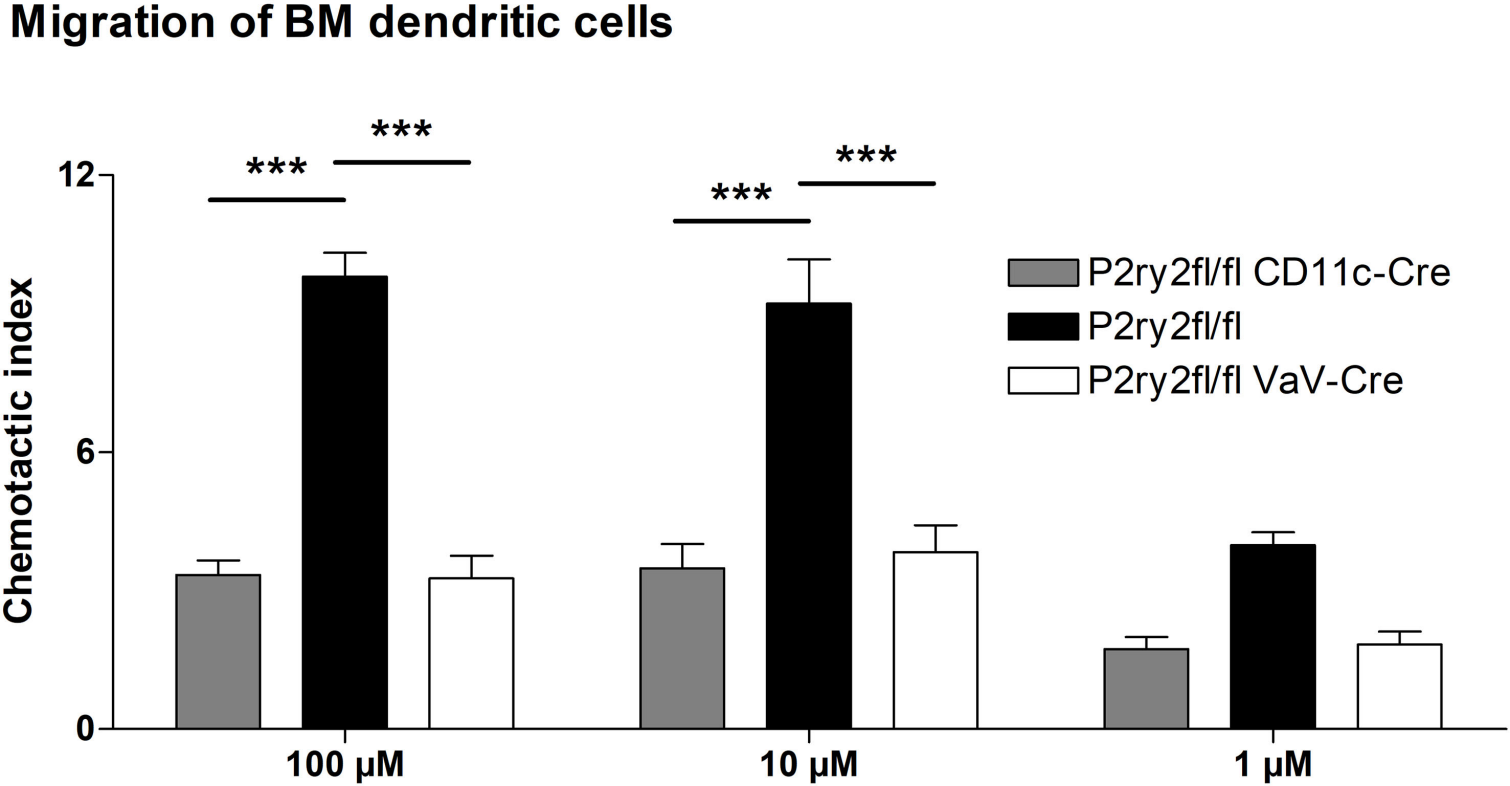
Dendritic cell migration towards ATP is reduced. Migratory capacity of BM-derived dendritic cells from *Cd11c-cre^+^* and *Vav-cre^+^* mice compared to cre-negative littermates. Graphs show mean ± SEM (n = 3). ***p < 0.001.

Next, we analyzed the effect of deletion of *P2ry2* in hematopoietic cells using Vav-Cre transgenic mice expressing Cre recombinase under the control of the human vav 1 oncogene (VAV1) regulatory elements in all hematopoietic stem cell. Ablation of *P2ry2* in hematopoietic cells in *Vav*-cre^+^ mice was accompanied by alleviated BALF eosinophils (**Figure 2A**) and a reduction in AAI marker cytokines (**Figure 2B**) after HDM challenge compared to cre-negative *P2ry2^fl/fl^* littermates. Histological analysis of the lung sections indicated lower peribronchial inflammation in *P2ry2^fl/fl^ Vav*-cre^+^ mice compared to that of *P2ry2^fl/fl^* mice after HDM-challenge (**Figure 2C**). There was no difference in sVCAM1-levels measured in BALF supernatants (**Figure 2D**). Changes in airway resistance in response to increasing doses of inhaled methacholine was measured, and the results revealed a decreased BHR in *P2ry2^fl/fl^ Vav-*cre^+^ mice (**Figure 2E**). Furthermore, we detected alleviated migration of BM dendritic cells from both *Vav*-cre^+^ and *Cd11c*-cre^+^ towards different concentrations of ATP compared to cre-negative littermates (**Figure 3**). These findings show that *P2ry2*-deficient mice in hematopoietic cells demonstrate an attenuated AAI phenotype.

### 3.3 Genetic *P2ry2* ablation in myeloid cells reduces AAI

**Figure 4:**
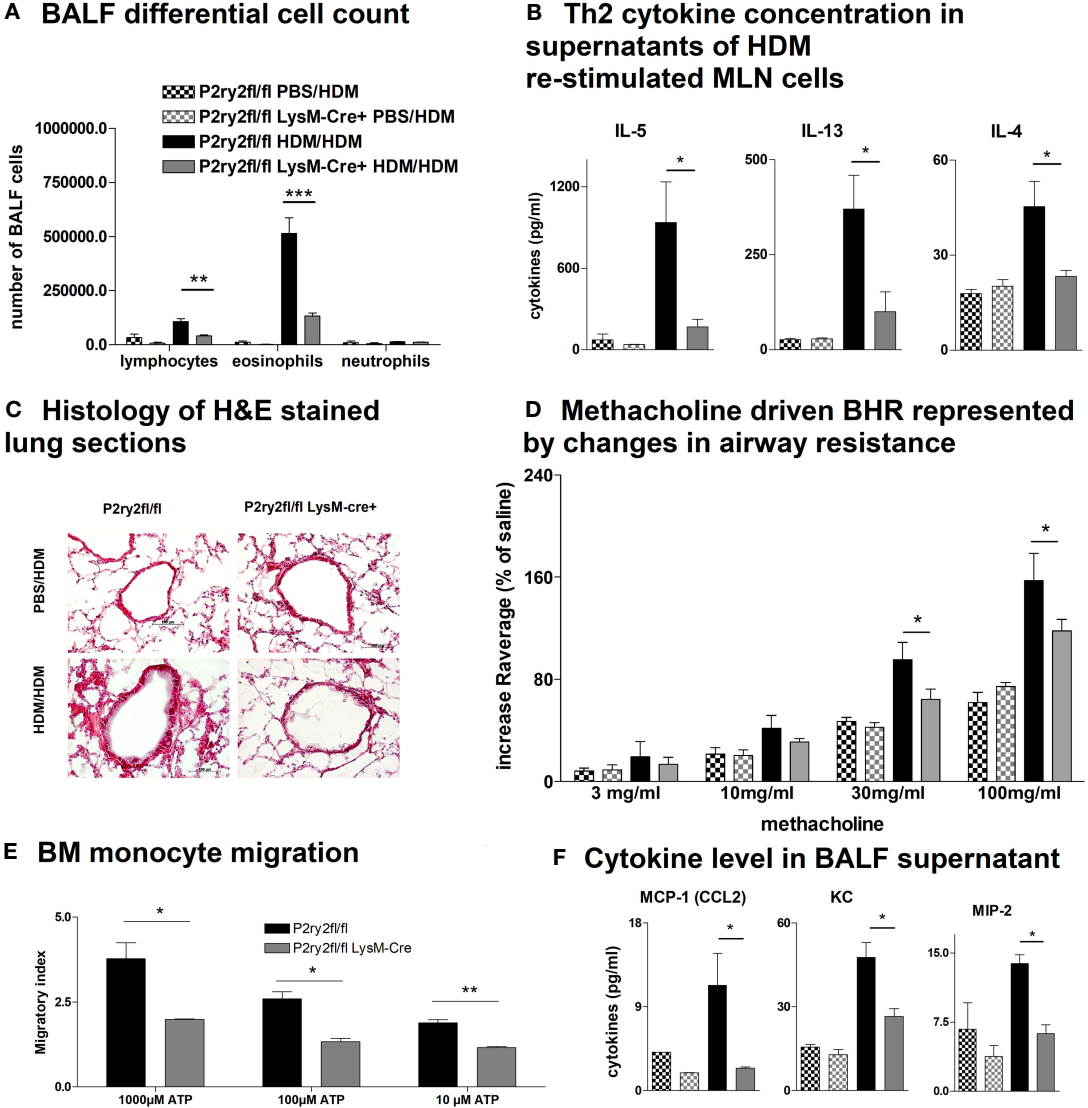
Ablation of *P2ry2* in *P2ry2^fl/fl^ LysM*-cre^+^ diminishes AAI. **(A)** Total number of neutrophils, eosinophils, and lymphocytes. **(B)** Concentration of Th2 in supernatants of MLN cells after HDM re-stimulation. **(C)** Histological H&E staining from lungs taken from *P2ry2^fl/fl^ LysM*-cre^+^ mice and *P2ry2^fl/fl^* mice after HDM challenge (magnification: 20× objective). **(D)** Changes in airway resistance in response to increasing doses of inhaled methacholine. **(E)** Chemotactic index of bone-marrow-derived monocytes compared to cre-negative littermates. **(F)** Levels of CCL2, KC, and MIP-2 in BALF supernatant. Graphs show mean ± SEM (n = 4–6). *p < 0.05, **p < 0.01, ***p < 0.001, *P2ry2^fl/fl^ LysM-cre^+^* HDM/HDM vs. *P2ry2^fl/fl^* HDM/HDM.

**Figure 5:**
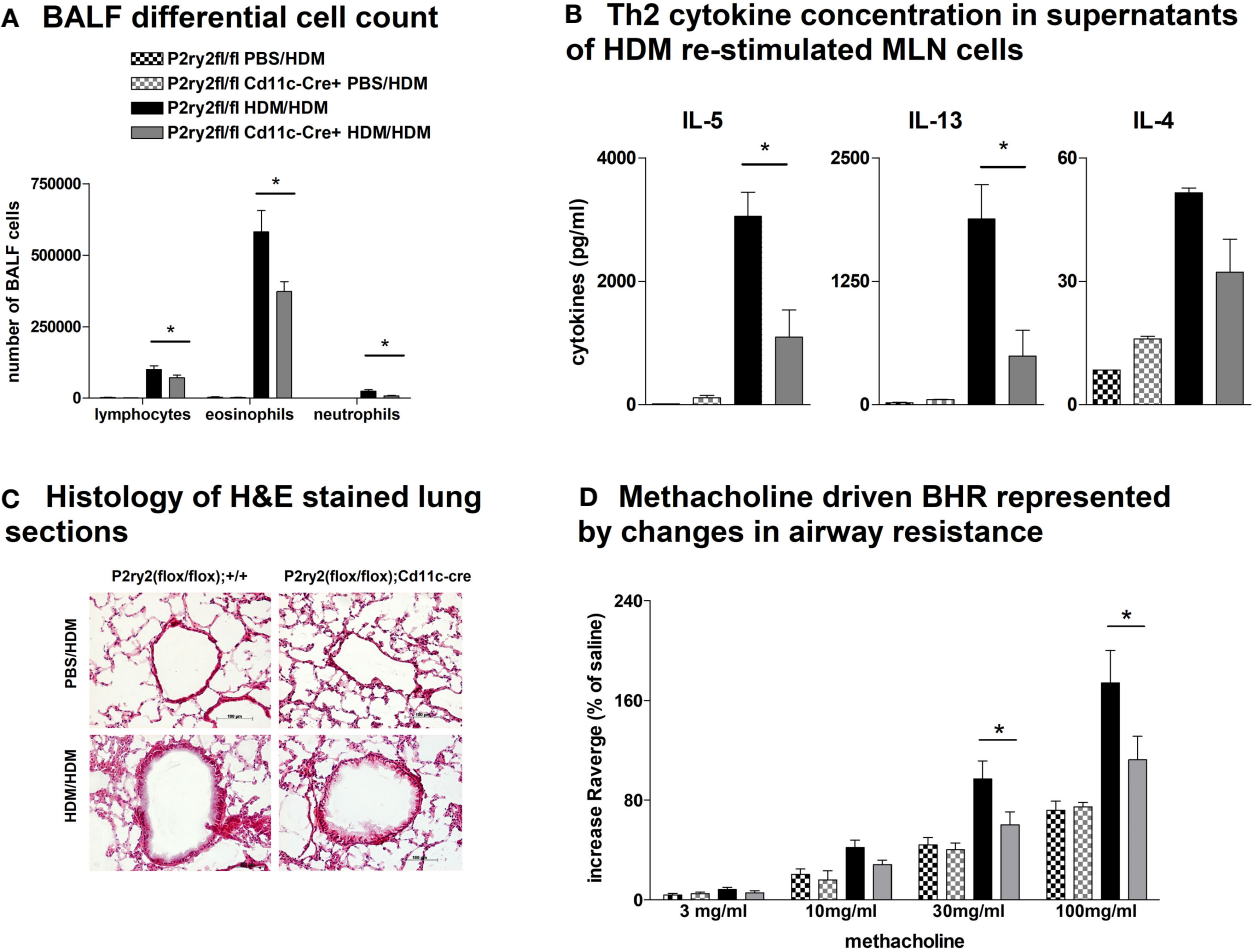
Recombinase activity in *P2ry2^fl/fl^ Cd11c-*cre^+^ mitigates AAI. **(A)** Differential cell count of BALF. **(B)** IL-4, IL-5, and IL-13 concentrations in supernatants of HDM re-stimulated MLN cells. **(C)** Histology of H&E-stained lung sections obtained from *P2ry2^fl/fl^ Cd11c*-cre^+^ mice and *P2ry2^fl/fl^* mice challenged with HDM (magnification: 20× objective). **(D)** BHR in response to increasing doses of inhaled methacholine, analyzed by recording the changes in airway resistance. Graphs show mean ± SEM (n = 4–6). *p < 0.05, *P2ry2^fl/fl^ Cd11c-cre^+^* HDM/HDM vs. *P2ry2^fl/fl^* HDM/HDM.

To elucidate whether the AAI reduction in *P2ry2^fl/fl^ Vav*-cre^+^ mice was conveyed by monocytes and macrophages, we performed experiments with *P2ry2^fl/fl^ LysM-*cre^+^ mice missing *P2ry2* in myeloid cells. Consistently, we noticed extenuated AAI indicated by reduced BALF eosinophilia (**Figure 4A**) and decreased production of Th2-cytokines by HDM re-stimulated MLN cells (**Figure 4B**). In line with these data, histological analysis of the lungs showed diminished peribronchial inflammation in *P2ry2^fl/fl^ LysM*-cre^+^ mice compared to *P2ry2^fl/fl^* mice after HDM challenge (**Figure 4C**). By measuring airway resistance, we observed reduced BHR to increasing doses of inhaled methacholine in *LysM*-cre^+^ mice compared to *P2ry2^fl/fl^* mice (**Figure 4D**). Moreover, we observed shortened migration of BM-derived monocytes from *LysM-*cre^+^ mice towards various levels of ATP in *in vitro* experiments (**Figure 4E**). In addition, we detected reduced levels of chemokine (C–C motif) ligand 2 (CCL2) and the murine IL-8 homologues KC and MIP-2 in BALF supernatants (**Figure 4F**). In *P2ry2^fl/fl^ Cd11c-*cre^+^ mice, we observed an alleviated AAI including BALF eosinophilia (**Figure 5A**) and production of Th2-cytokines by HDM re-stimulated MLN cells (**Figure 5B**). Histological analysis of the lung sections obtained from *P2ry2^fl/fl^ Cd11c*-cre^+^ mice and *P2ry2^fl/fl^* mice challenged with HDM confirmed reduced inflammatory cell recruitment in the lungs of *P2ry2^fl/fl^ Cd11c*-cre^+^ mice (**Figure 5C**). Measurement of airway resistance to increasing doses of inhaled methacholine revealed reduced BHR in *P2ry2^fl/fl^ Cd11c*-cre^+^ mice in comparison to *Cd11c*-cre^−^ littermates (**Figure 5D**). Taken together, these results indicate that *P2ry2* ablation in myeloid cells reduces AAI.

### 3.4 Mice lacking *P2ry2* in CD4^+^ cells present unchanged AAI

**Figure 6:**
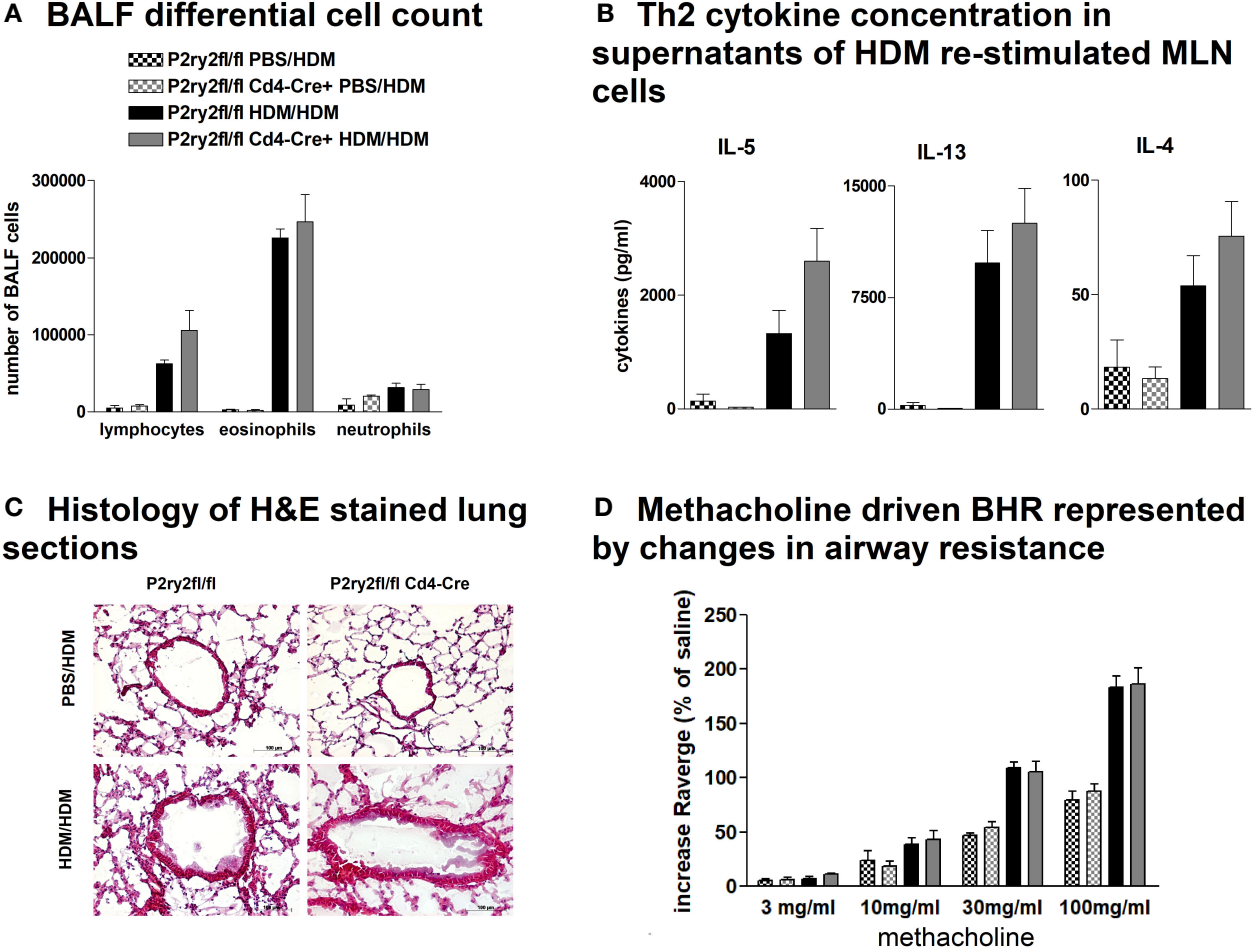
*P2ry2* deficiency in *P2ry2^fl/fl^ Cd4*-cre^+^ does not affect degree of AAI. **(A)** Total count of neutrophils, eosinophils, and lymphocytes. **(B)** Concentration of Th2 determined in supernatants of MLN cells after HDM re-stimulation. **(C)** Histology of H&E-stained lung sections (magnification: 20× objective) taken from *P2ry2^fl/fl^ Cd*4-cre^+^ mice and *P2ry2^fl/fl^* mice after HDM challenge. **(D)** Differences in airway resistance in response to increasing doses of inhaled nebulized methacholine. Graphs show mean ± SEM (n = 4–6). *P2ry2^fl/fl^ Cd4*-cre^+^ HDM/HDM vs. *P2ry2^fl/fl^* HDM/HDM.

To investigate the role of *P2ry2* in CD4^+^ cells, we created conditional knockout mice deficient in the P2RY2 receptor in T cells. *P2ry2^fl/fl^ Cd4-*cre^+^ mice did not show different hallmarks of AAI compared to their *Cd4*-cre^−^ littermates. We did not observe a reduction in BALF eosinophilia (**Figure 6A**) and production of Th2-cytokines by HDM re-stimulated MLN cells (**Figure 6B**). Consistently, histological screening of stained lung sections taken from *P2ry2^fl/fl^ Cd4*-cre^+^ and *P2ry2^fl/fl^* mice after HDM challenge indicated no difference in inflammation between treatments in different mice groups (**Figure 6C**). As expected, measuring airway resistance also indicated no changes in BHR to increasing doses of inhaled methacholine (**Figure 6D**). Taken together, these data suggest that *Cd4*-cre^+^ *P2ry2^fl/fl^* mice deficient in *P2ry2* in CD4-positive cells exhibit unchanged AAI compared to their cre-negative littermates.

## 4 Discussion

We have previously shown that *P2ry2* deficiency is accompanied by an attenuated acute ovalbumin-induced airway inflammation (25). However, the OVA model begins with a systemic sensitization caused by injecting OVA coupled with aluminum hydroxide, which activates the NACHT, LRR, and PYD domains-containing protein 3 (NALP3) inflammasome and subsequently drives the activation of antigen presentation cell (APC) (63). Thus, this model has difficulty in simulating the human situation, in which sensitization occurs by pattern-recognition receptors (PRRs) ligand binding on the airway epithelium (64, 65). In this study, we used an HDM-based model of allergic airway inflammation, by administering house dust mite extract intratracheally to mice, which better imitates the human sensitization by taking the way through the airways including immediate innate immune response (66). Notably, the HDM-mouse model showed increased pulmonary ATP levels (67). Extracellular ATP accumulates in the lungs of asthmatic patients and animals in experimental models of allergic airway inflammation (52). In these settings, we previously observed that interference with ATP/type 2 purinergic receptor pathways attenuates allergic airway inflammation (25, 52, 53). We focused on investigating the cell-specific effects of P2RY2 signaling by applying conditional knockout animals. In these models, P2RY2 is inactivated in specific cell types such as alveolar epithelial cells, hematopoietic cells, myeloid cells, or dendritic cells, and CD4^+^ cells. We used the Cre-lox system, in which Cre recombinase recognizes the loci of recombination loxP that causes a deletion of the P2RY2 between the two loxP sites in a cell-specific manner. Other cell types and tissues exhibit an unmodified, functional P2RY2 expression. We identified for the first time that airway epithelial cells, monocytes, and dendritic cells express P2RY2 mediate acute airway inflammation in the HDM mouse model. We found that knockout of *P2ry2* in these models resulted in reduced BALF eosinophilia, attenuated type 2 cytokine production of HDM re-stimulated MLN cells, decreased peribronchial inflammation, and reduced bronchial hyperreactivity in response to increasing doses of nebulized methacholine.

Clara cells represent a pool of non-ciliated secretory cells in the small airways and trachea, where they contribute to the control of inflammation and maintenance of the ciliated airway epithelial cell population (68). Activation of airway epithelial cells strongly depends on PAMPs, DAMPs, and their corresponding receptors (69). Former studies implicated autocrine ATP-induced P2RY2 signaling in mucociliary clearance (38) and IL-33 release by airway epithelial cells (70). To establish an HDM-induced model of airway inflammation, we applied HDM intratracheally to *P2ry2^fl/fl^ Cct*-cre^+^ mice, which are deficient for *P2ry2* in the epithelial cells of the proximal conducting airways. Importantly, we found that *P2ry2^fl/fl^ Cct*-cre^+^ mice showed an attenuated BALF eosinophilia, type 2 cytokine production of HDM re-stimulated MLN cells, peribronchial inflammation, and BHR in response to increasing doses of nebulized methacholine compared to cre-negative littermates. This alleviated AAI phenotype observed in *P2ry2^fl/fl^ Cct*-cre^+^ mice is in line with previous findings describing a contribution of P2RY2 signaling to IL-33 secretion by airway epithelial cells subsequently initiating a type 2 immune response (70). Consequently, *P2ry2^fl/fl^ Cct*-cre^+^ mice exhibited diminished IL-33 levels in the lung homogenates and at mRNA level compared to cre-negative littermates. Remarkably, we have noticed that epithelial cells appeared small and disorganized in *P2ry2^fl/fl^ Cct*-cre^+^ mice, which might be due to the effect of the deletion. To our knowledge, there is no description of this phenomenon yet, but it should be investigated further.

In addition to epithelial cells playing a crucial role in initiating inflammatory immune responses against allergens, cells of hematopoietic origin drive inflammation by exerting its specific effector functions (11). P2RY2 expression has been reported in monocytes, macrophages, dendritic cells, neutrophils, eosinophils, NK cells, and lymphocytes (11, 42), where it contributes to migration and mediator production (12, 25, 30). Consistently, the genetic ablation of *P2ry2* in hematopoietic cells in *P2ry2^fl/fl^ Vav*-cre^+^ mice results in an alleviated HDM-induced airway inflammation in terms of eosinophils in the BALF, production of type 2 cytokine of HDM re-stimulated MLN cells, peribronchial inflammation, and BHR compared to cre-negative littermates.

Dendritic cells (DCs) are essential for initiation and maintenance of Th2 response to inhaled allergens in asthma pathogenesis (71). DCs can derive from common dendritic cell progenitors (CDPs) subsequently infiltrate secondary lymphoid organs and tissue or differentiate from monocytes (72). Previously, our group reported an alleviated AAI phenotype in *P2ry2* knockout mice applied to an OVA-driven model, which was associated with a reduced ATP-mediated chemotactic capacity of dendritic cells (25). Likewise, BMDCs derived from *P2ry2^fl/fl^ Vav*-cre^+^ mice exhibited a reduced *in vitro* migration towards ATP compared to BMDCs from cre-negative littermates. Beside hematopoietic cells, *P2ry2^fl/fl^ VaV*-cre^+^ mice exhibit *P2ry2* deficiency in endothelial cells. Vanderstocken et al. demonstrated that *P2ry2* knockout mice produce less vascular cell adhesion molecule 1 (VCAM-1) in an OVA-driven model of airway inflammation resulting in a mitigated eosinophil adhesion and infiltration (73). Nevertheless, we did not observe reduced sVCAM1 levels in the BALF of *P2ry2^fl/fl^ VaV*-cre^+^ mice compared to cre-negative littermates. Thus, P2RY2 signaling is implicated in mediating its proinflammatory effects on cells of hematopoietic origin rather than endothelial cells in our HDM-induced model of airway inflammation.

Further confirmation came from *P2ry2^fl/fl^ Cd11c*-cre^+^ and *P2ry2^fl/fl^ LysM*-cre^+^ mice, which also exhibited an alleviated asthma phenotype including attenuated BALF eosinophilia, type 2 cytokine secretion by HDM re-stimulated MLN cells, peribronchial inflammation, and BHR similar to *P2ry2^fl/fl^ Vav*-cre^+^ animals. In addition, BM-derived dendritic cells isolated from *P2ry2^fl/fl^ Cd11c*-cre^+^ and BM-derived monocytes isolated from *P2ry2^fl/fl^ LysM*-cre^+^ showed an attenuated *in vitro* migration in response to ATP compared to their cre-negative littermates. This is in line with previous reports demonstrating that P2RY2 contributes to allergen-induced airway inflammation by driving myeloid dendritic cell and monocytes lung recruitment (25, 29, 52, 74). Furthermore, P2RY2 on monocytes and macrophages is implicated in the production of the chemoattractant MCP-1/CCL2, which attracts dendritic cells, monocytes, and T cells towards the sides of inflammation (75–77). Concordantly, we found reduced MCP-1/CCL2 and murine IL-8 homologues KC and MIP-2 levels in the BALF of *P2ry2^fl/fl^ LysM*-cre^+^ mice compared to cre-negative littermates. Beside MCP-1/CCL2, *P2ry2^fl/fl^ LysM*-cre^+^ mice also exhibit diminished levels of the murine IL-8 homologues KC and MIP-2 in BALF corresponding with former observations that P2RY2 on monocytes is required for proinflammatory IL-8 secretion (78). Next, to investigate the role of *P2ry2* in CD4^+^ cells, we generated conditional knockout mice lacking *P2ry2* receptor in T cells. We found that *P2ry2* in CD4^+^ cells is not essential for controlling CD4^+^ response during AAI in HDM mouse model.

Regarding the correlation of different types of involved cells, a remarkable array of defense mechanisms is implemented by the mammalian immune system to fight infections (79). Hematopoietic immune cells, such as myeloid cells that regulate innate immunity and lymphoid cells that make up adaptive immunity, are its primary constituents (79). There is structural immunity, which is the term used for the immune functions in the non-hematopoietic, structural cell populations of the body such as epithelial cells (79). It has been previously shown that the Th2 inflammatory responses are characterized by the recruitment and activation of mast cells, basophils, and eosinophils in airway and intestinal epithelia (80, 81). Furthermore, we have previously demonstrated that P2RY2 signal transduction in immune cells influences the pathogenesis of various inflammatory airway diseases (74). Thus, epithelial cells and myeloid cells might be involved in the inflammation and immune responses by different mediators directed in non-hematopoietic and hematopoietic pathways, respectively. In this context, purinergic receptors such as P2X and P2Y play a fundamental role in mediating lung inflammation associated with asthma and COPD.

This study proposes P2RY2 as a key player in HDM-induced airway inflammation. The P2RY2 signal transduction has a highly ambivalent effect in the body and can have a protective effect by defending against bacterial infections, promoting wound healing or the stimulation of mucociliary clearance, but at the same time, it can promote the pathogenesis of chronic inflammatory diseases. Therefore, P2RY2 antagonists are now also being considered as a therapeutic option, for example for the treatment of inflammatory respiratory diseases such as asthma or COPD (11). The studies by Müller et al. already provided the first evidence for the involvement of the P2Y2 receptor in allergic airway inflammation and its potential suitability as a target in the therapy of asthma bronchiales (25). The present work contributes to a better understanding of cells that mediate proinflammatory effects in allergic asthma via P2RY2 signaling. This provides new insights into the asthma pathogenesis and offers new possibilities regarding a cell-specific therapy of allergic asthma by P2RY2-targeted medication. In this respect, the accessibility of the airway epithelium, at the interface of the external and internal milieu, opens new avenues for the development of a new generation of potential therapies, such as RNA-based therapies, which could be administered via inhalation for rapid and targeted uptake by epithelial cells directly involved in driving airway inflammation. Epithelial miRNAs targeting P2RY2 are highly attractive for therapeutic intervention, since the airway epithelium is present and enables delivery of therapeutic drugs via an inhaled route. Non-epithelial cells in the airways, especially macrophages and other phagocytic cells, may also take up therapeutic oligonucleotides. However, studies using fluorescently conjugated antagomirs in mice have demonstrated efficient uptake specifically by the airway epithelium compared to other cellular populations (82). Using miRNA reporter systems to carefully target P2RY2 in epithelial cells will be an important future goal.

Taken together, our findings clearly demonstrate that P2RY2 promotes HDM-induced airway inflammation by mediating proinflammatory cytokine production in airway epithelial cells, monocytes, and dendritic cells. P2RY2 can also promote lung recruitment of dendritic cells and monocytes. In addition, P2RY2 on the epithelium favors IL-33 expression and subsequent Th2-like immune response in the airways. That points towards an involvement of the innate immune system in asthma pathogenesis. Our investigations concerning bone-marrow-derived cells from hematopoietic origin support our former findings demonstrating that P2RY2 is crucial for migration and maintenance of asthma by recruitment of dendritic cells. More unexpected was the apparent participation of monocytes/macrophages in asthma conducted possibly by production of MCP-1/CCL2, IL-8 homologues, and recruitment of monocytes into the lungs.

In conclusion, we previously described the potential role of P2RY2 in AAI, and in this study, we further identified the cell-type-specific role of P2RYR in AAI. Our results emphasize the importance of the P2Y2 receptor signaling specifically expressed by airway epithelial cells, monocytes, and dendritic cells as a potential therapeutic target in AAI. These findings point to the diversity and ambivalence of immune modulatory effects induced by P2RY2 signaling and underlines the necessity for further investigations.

## Data availability statement

The original contributions presented in the study are included in the article/**Supplementary Material**, further inquiries can be directed to the corresponding author/s.

## Ethics statement

Mice experiments were performed in accordance to the local ethic committee (G12-096) of Freiburg University. The study was conducted in accordance with the local legislation and institutional requirements.

## Author contributions

DS and AE-G provided the main manuscript. AE-G prepared the revised manuscript and point-by-point response to reviewers’ comments. DS performed the mice experiments. DS and MI performed data analysis and statistical analysis. MI performed lung function analysis. ZK and AE-G generated BM-derived macrophage, monocytes, and dendritic cells. MK performed genotyping and breeding of animals. SM and SS assisted with *in vitro* experiments. MI planned and supervised the study. All authors contributed to the article and approved the submitted version.
