# Supplementary material for: Cell-type-specific role of P2Y2 receptor in HDM-driven model of allergic airway inflammation
Source: Front Immunol. 2023 Sep 14;14:1209097. doi: 10.3389/fimmu.2023.1209097 (PMC10543084; doi:10.3389/fimmu.2023.1209097)

Supplementary Material

1 Supplementary Data

qPCR for confirmation of correct recombinase activity

To confirm correct genotyping and recombinase activity, qPCR for *P2ry2* expression in different sorts of cells was carried out. We observed relative reduction of *P2ry2* expression in lung epithelial cells (EpCAM+, CD45-, CD31-) obtained from *P2ry2*$^{fl/fl}$ *CCt*-cre+ and *P2ry2*$^{fl/fl}$ in conditional mice compared to controls (Supplementary Fig. 1*A*). Concordant, a lower expression of *P2ry2* was also measured in BMDM- and BM- derived monocytes stimulated with LPS obtained from *P2ry2*$^{fl/fl}$ *LysM*-cre^{+} mice compared to *P2ry2*$^{fl/fl}$ (Supplementary Fig. 1*B* and *D*). *P2ry2* mRNA was not detectable in PBMCs and granulocytes from *P2ry2*$^{fl/fl}$ *Vav*-cre^{+} mice compared to *P2ry2*$^{fl/fl}$ mice. We observed a strong reduction of *P2ry2* mRNA levels in LPS-stimulated PBMCs and granulocytes from *P2ry2*$^{fl/fl}$ *LysM*-cre^{+} mice compared to a high expression in *P2ry2*$^{fl/fl}$ mice (Supplementary Fig. 1*C* and *E*). CD11c^{+} lung cells collected from *P2ry2*$^{fl/fl}$ *Cd11c*-cre^{+} mice compared to *P2ry2*$^{fl/fl}$ mice showed a significant reduction of *P2ry2* expression in *P2ry2*$^{fl/fl}$ *Cd11c*-cre+ mice (Supplementary Fig. 1*F*). These data further revealed a reduction of *P2ry2* expression in BM-derived dendritic cells from *P2ry2*$^{fl/fl}$ *Cd11c*-cre^{+} mice compared to the *P2ry2*$^{fl/fl}$ mice (Supplementary Fig. 1*C*). Subsequently, we observed a reduced expression of *P2ry2* in CD4^{+} cells gained from the spleen of *P2ry2*$^{fl/fl}$ *Cd4*-cre^{+} mice compared to *P2ry2*$^{fl/fl}$ mice (Supplementary Fig. 1*H*).

2 Supplementary Figures

Supplementary figure 1

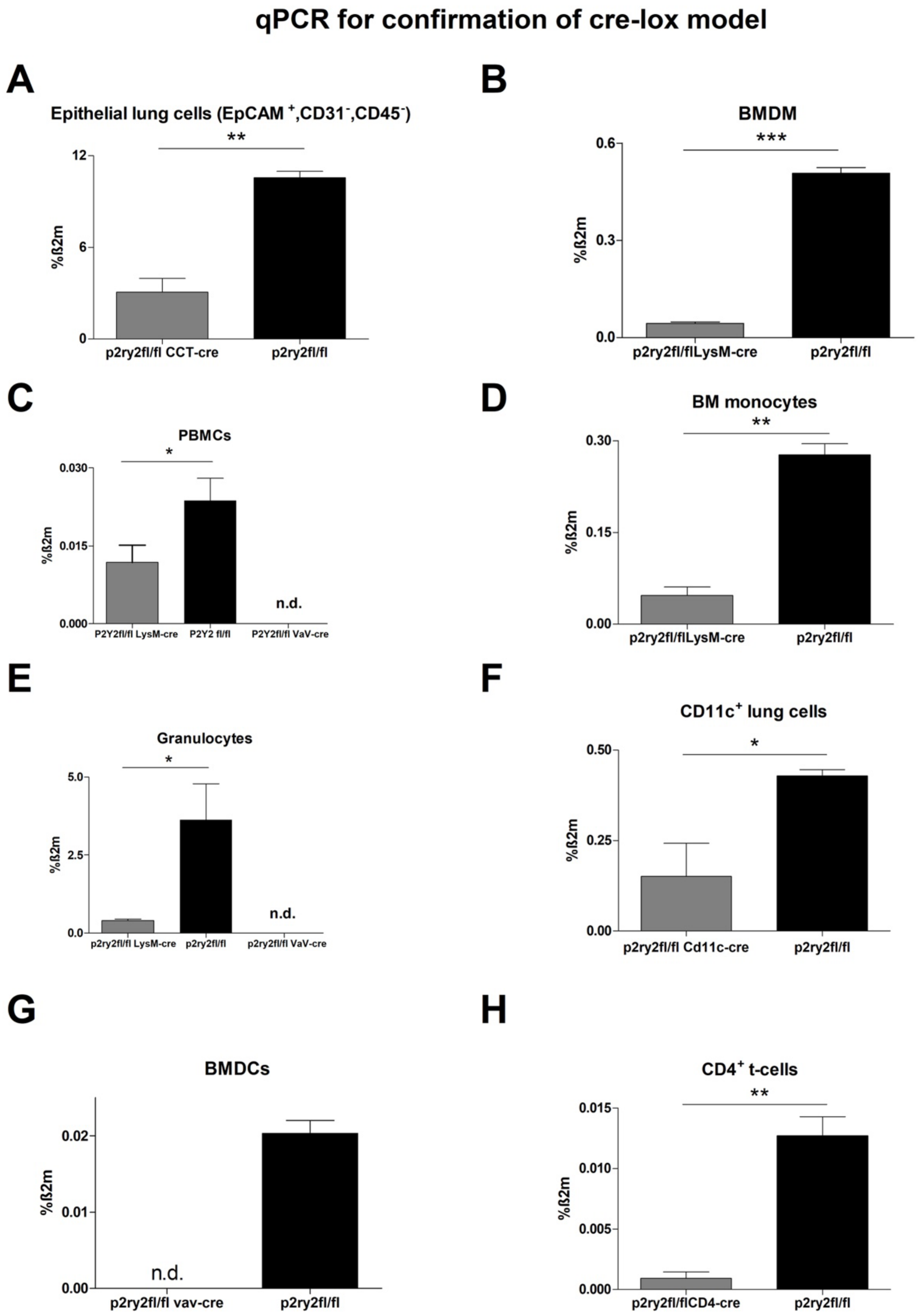

Supplementary figure 1. Confirmation of the genotype. ***A)*** Relative *P2ry2* expression in FACS sorted epithelial cells (EpCAM$^+$, CD45$^-$, CD31$^-$) obtained from lung digest from *P2ry2$^{fl/fl}$ CCt*-cre$^+$ and *P2ry2$^{fl/fl}$* lungs. ***B)*** Relative expression of *P2ry2* in BMDM stimulated with LPS obtained from *P2ry2$^{fl/fl}$ LysM*-cre$^+$ mice and *P2ry2$^{fl/fl}$*. ***C)*** Relative *P2ry2* expression

in PBMC from *P2ry2*$^{fl/fl}$ *Vav*$^{+}$, *P2ry2*$^{fl/fl}$ *LysM*$^{+}$ and *P2ry2*$^{fl/fl}$ mice. ***D)*** Relative *P2ry2* expression in BM-derived monocytes from *P2ry2*$^{fl/fl}$ *LysM*$^{+}$ and *P2ry2*$^{fl/fl}$ mice. ***E)*** *P2ry2* expression in granulocytes isolated from peripheral blood from *P2ry2*$^{fl/fl}$ *Vav*$^{+}$ mice, *P2ry2*$^{fl/fl}$ *LysM*-cre^{+} mice and *P2ry2*$^{fl/fl}$. ***F)*** Relative *P2ry2* mRNA level in CD11c^{+} cells collected from *P2ry2*$^{fl/fl}$ *Cd11c*-cre^{+} mice and *P2ry2*$^{fl/fl}$. Isolated from lung digest using magnetic cell separation. ***G)*** *P2ry2* expression in BM-derived DCs from *P2ry2*$^{fl/fl}$ *Cd11c*-cre^{+} mice and cre negative littermates. ***H)*** Relative *P2ry2* mRNA level in CD4^{+} cells collected from *P2ry2*$^{fl/fl}$ *Cd4*-cre^{+} mice and *P2ry2*$^{fl/fl}$ mice spleen digest using magnetic cell separation. *P2ry2* expression was determined using quantitative Real Time-PCR. Graphs show mean ± SD (n =3). * $P < 0.05$, ** $P < 0.01$, *** $P < 0.001$. mice: *P2ry2*$^{fl/fl}$ cre+ and *P2ry2*$^{fl/fl}$.

3 Supplementary material and methods

3.1. House Dust Mite Acute (HDM) Mouse Model

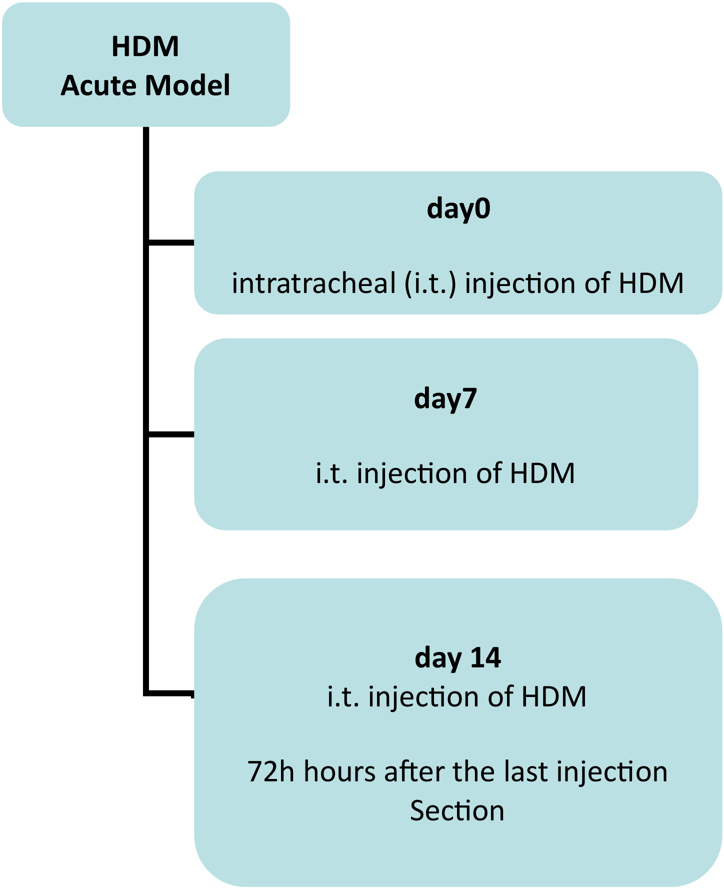

3.1.1. Material

- House Dust Mite (HDM, Greer Laboratories, Lenoir, NC)
  100 µg Dermatophagoides pteronyssinus extract dissolved in 80 µl PBS

3.1.2. Groups

Four groups were generated. Two interventional groups and two control groups. The interventional groups received HDM i.t at day 0, 7, and 14. The control groups received PBS i.t. at day 0 and 7 and HDM i.t. at day 14.

3.1.3. i.t. application

1. Inject C57BL/6 mice with anaesthesia intraperitoneally (i.p.) (Ketamin 100 mg/kg and Xylazin 5mg/kg), wait till the mice are asleep
2. Affix the mice at the i.t. appliance
3. Take the tongue outside with forceps, Importantly, the tongue must be outside, not inside, the mouth throughout the duration of the application.
4. Put 80µl of the treatment into the mouth and wait till you hear a stertorousness from the mice
5. Lay the mice onto the heating plate

3.1.4. H&E (Haematoxylin and Eosin) Staining for Frozen Tissue Sections

1. After euthanizing the mice, collect the BALF, remove the five lung lobes and embed them in Tissue-Tek O.C.T. and then freeze them in liquid nitrogen.
2. Section them into 5 µm slices, place on slides
3. Stain with filtered 0.1% Mayers Hematoxylin (Sigma; MHS-16) for 10-12 seconds
4. Rinse in cool running ddH_2O for 1 minute.
5. Immerse in 0,1% HCL water
6. Immerse in undiluted Eosin for 10-12 seconds
7. Rinse in cool running ddH_2O
8. Dehydrate in 70% EtOH for 1 minute
9. Dehydrate in 95% EtOH for 1 minute.
10. Place in xylene for 1 minute, repeat in fresh xylene
11. Clean slide off with a kimwipe; mount and coverslip with Entellan new (Sigma-Aldrich, Deisenhofen)

3.1.5. Statistical analysis

For the calculation of the statistical significance of differences between groups, one-way ANOVA was applied, followed by Bonferoni comparison test. Analyses were performed using Prism v8 (GraphPad Software, La Jolla, CA, USA). Differences were considered significant at $P < 0.05$.

3.2. Identification of Lymphocytes, Eosinophil, and Neutrophil in BALF using flow cytometry.

1- Set up the primary gates based on the forward and side scatter, excluding debris

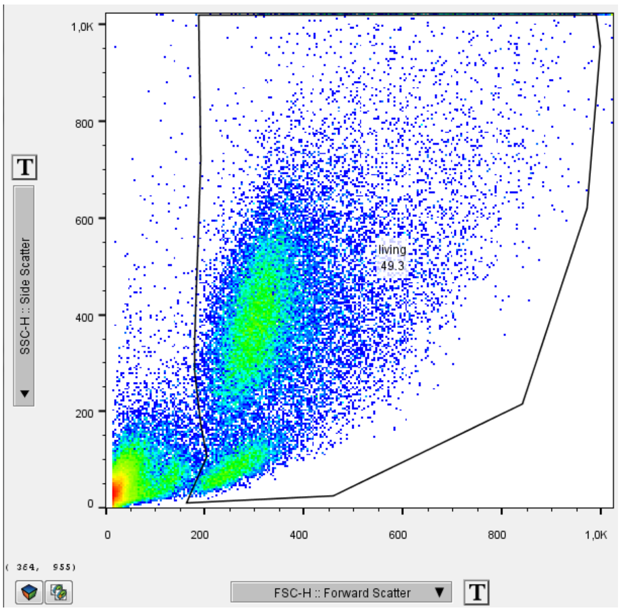

2- Set up the gate on live cells
3- For Lymphocyte gate
   A) Set up the gate on SSC – FSC

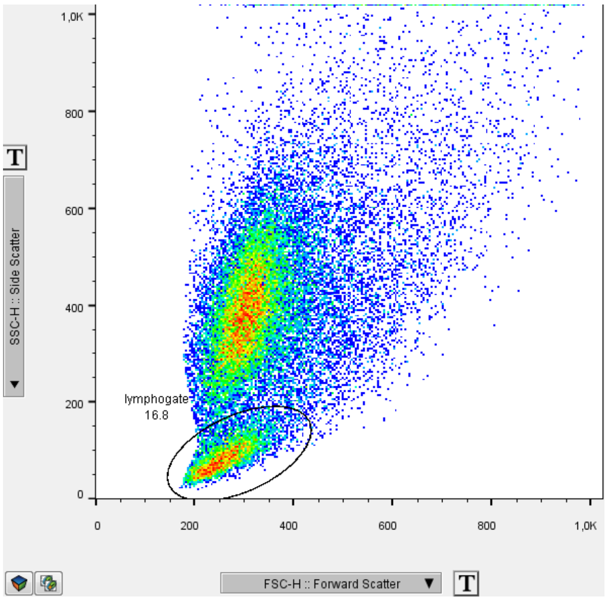

B) Gate B220+ CD3+ (T- and- B-Lymphocytes): CD3+/B220 - FSC

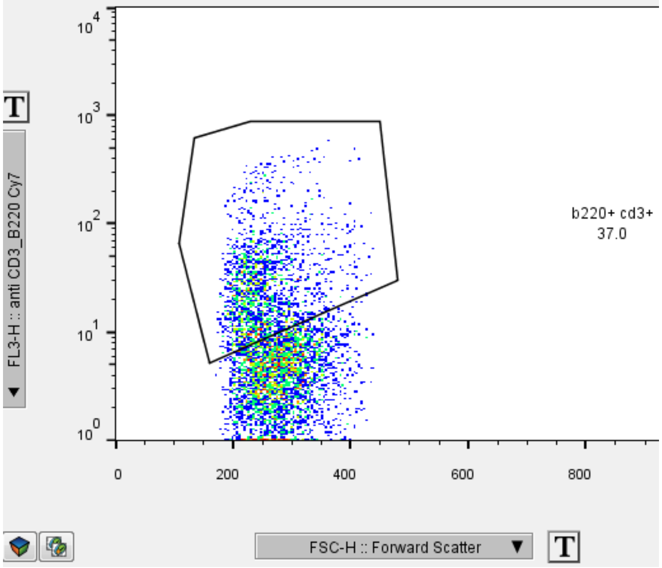

C) Gate lymphocytes: SSC -FSC

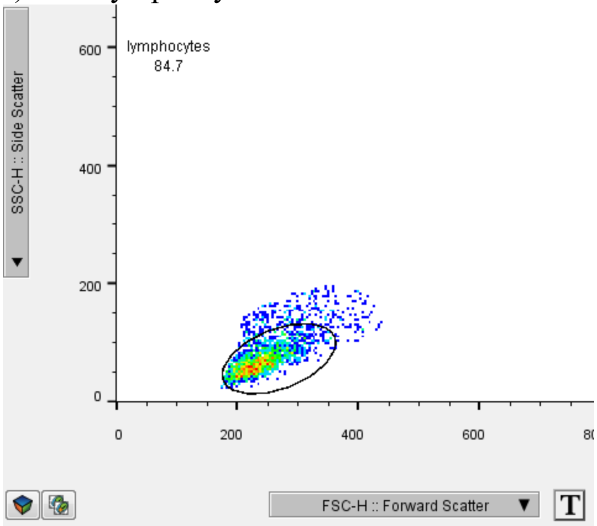

3. Eosinophil/Neutrophil

Set up the gate on:

A) CD3-/B220-: CD3/B220 – FSC

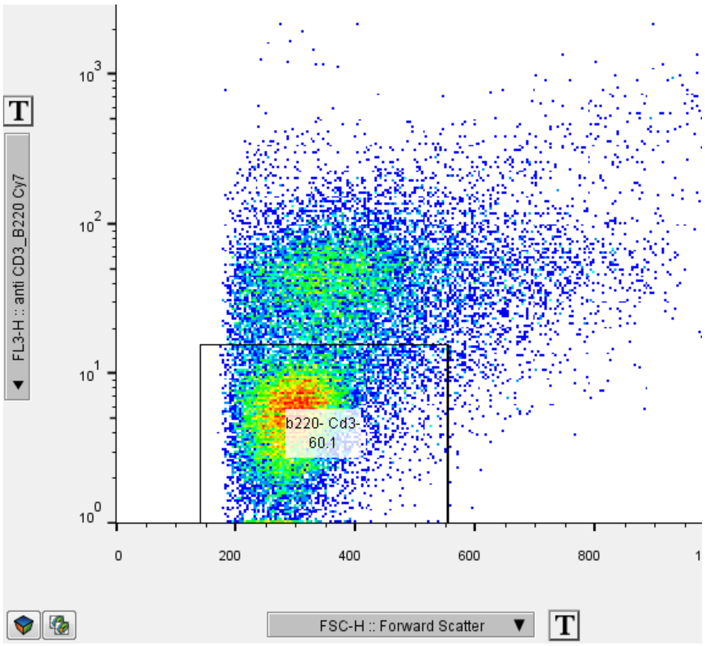

B) CD11c-: CD11c - FSC

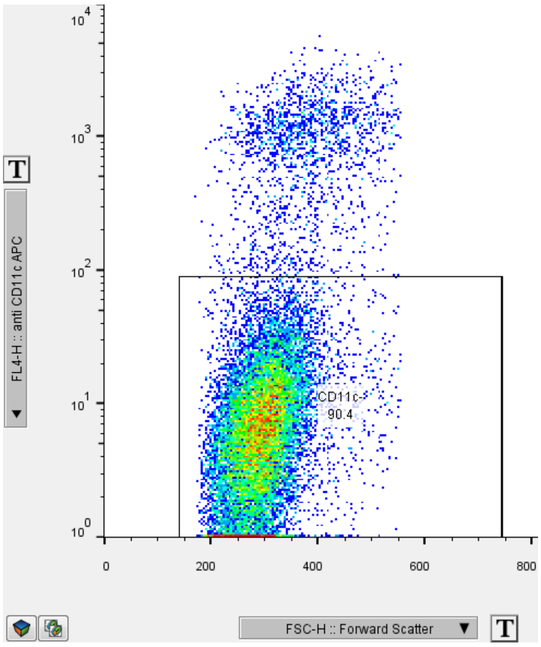

C) CCR3+-> Eosinophil; GR1+-> Neutrophil: CCR3 – GR1

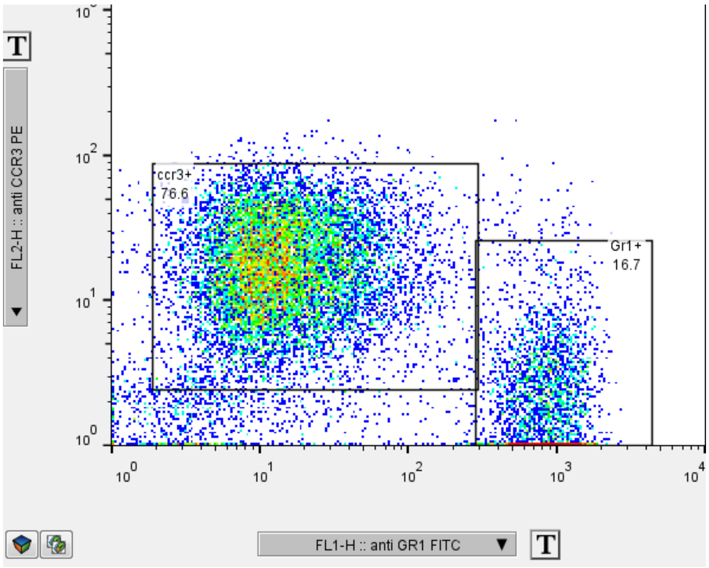

D) Eosinophil (CCR3)

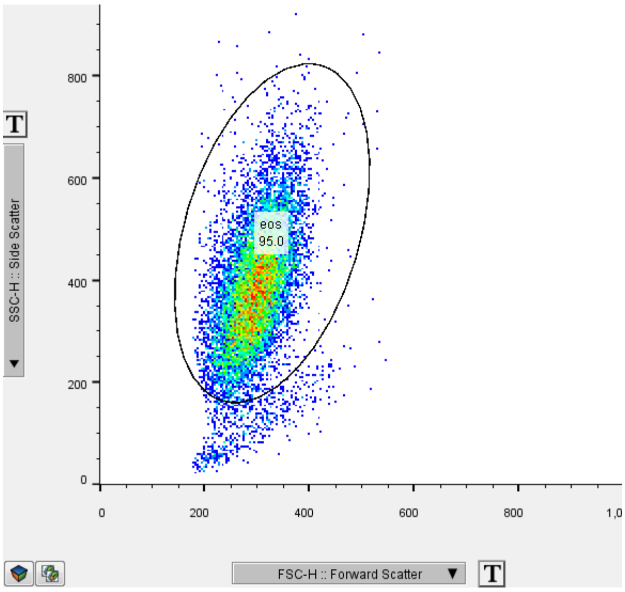

Neutrophil (GR1+)

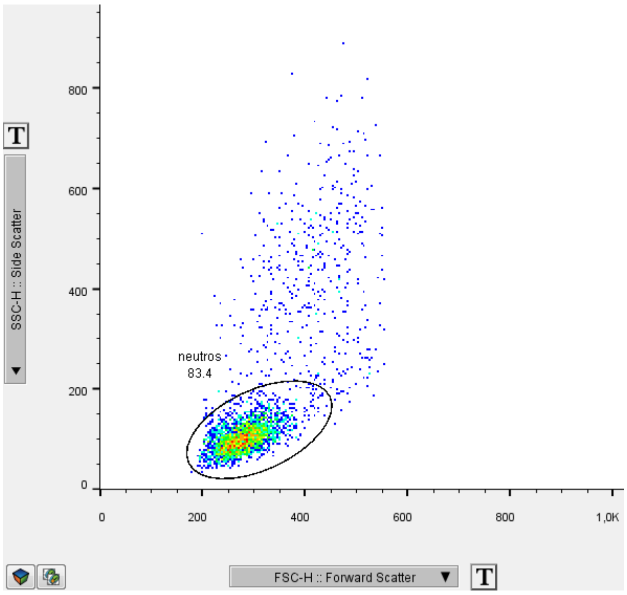

Supplement: Supplementary file 1 [file DataSheet_1.pdf]
